# Hemostasis pad combined with compression device after transradial coronary procedures: A randomized controlled trial

**DOI:** 10.1371/journal.pone.0181099

**Published:** 2017-07-24

**Authors:** Si-Hyuck Kang, Donghoon Han, Sehun Kim, Chang-Hwan Yoon, Jin-Joo Park, Jung-Won Suh, Young-Seok Cho, Tae-Jin Youn, In-Ho Chae

**Affiliations:** Division of Cardiology, Department of Internal Medicine, College of Medicine, Seoul National University and Cardiovascular Center, Seoul National University Bundang Hospital, Seongnam-si, Republic of Korea; Universita degli Studi Magna Graecia di Catanzaro, ITALY

## Abstract

**Background:**

Arterial access and hemostasis are important processes during percutaneous coronary procedures. In this study, we tested if the use of chitosan-based pads on top of compression devices could improve hemostasis efficacy compared with compression devices alone after transradial coronary angiography or interventions.

**Methods:**

This study was a single-center open-label randomized controlled trial. Patients who underwent coronary angiography or intervention with the transradial approach were randomly assigned to the study (compression device and a chitosan-based pad) or control (compression devices alone) group in a 2:1 fashion. The primary endpoint was time to hemostasis, categorized into ≤5, 6–10, 11–20, and >20 minutes.

**Results:**

Between April and July 2016, 95 patients were enrolled (59 were assigned to the study arm and 36 to the control arm). Time to hemostasis, the primary endpoint, was significantly lower in the study group than in the control group (p<0.001). Both groups showed low rates of vascular complications.

**Conclusions:**

This study suggests that the use of a hemostasis pad in combination with rotatory compression devices is a safe and effective hemostasis strategy after radial artery access.

**Trial registration:**

ClinicalTrials.gov NCT02954029

## Introduction

The transradial approach is increasingly used for arterial access during percutaneous cardiovascular procedures. The main advantages of the transradial approach over the transfemoral approach include patient convenience, reduced time to hemostasis, a lower risk of acute kidney injury, and improved outcomes such as a lower risk of bleeding.[1, 2] However, radial access is still associated with significant complications such as access site bleeding and vessel occlusion.[3]

Effective and successful hemostasis is a key to reducing complications after coronary procedures.[4–6] Major bleeding after percutaneous coronary intervention is related to adverse outcomes.[7–9] On the other hand, overly aggressive hemostasis may cause radial artery occlusion [10]. Compression devices (CD) or hemostasis pads are popular methods for bleeding control after radial artery punctures.[11–13] In this study, we hypothesized that the combination of CD and chitosan-based pads would improve the hemostasis efficacy compared with CD alone after transradial coronary angiography or interventions.

## Methods

### Patients

This study was a prospective, single-center, open-label, randomized controlled trial designed to evaluate the safety and feasibility of the combined use of CD and hemostasis pads after transradial coronary procedures (S1, S2 and S3 Files). Patients aged ≥18 years who underwent elective or urgent coronary angiography or interventions with radial access were enrolled. Exclusion criteria were bleeding tendency, thrombocytopenia, and shellfish allergy. Study participants were recruited from the cardiovascular center of Seoul National University Bundang Hospital between April and July 2016. The Seoul National University Bundang Hospital institutional review board (IRB) approved this study protocol on Jan 11, 2016 (B1512-326-001), and all participants provided written informed consent. The authors confirm that all ongoing and related trials for this drug/intervention are registered (ClinicalTrials.gov ID: NCT02954029). The study was registered after the enrollment of participants began because of delays by the investigators.

### Treatment

Coronary angiography or intervention was performed per standard techniques. After gaining arterial access, the radial sheath was flushed with 5,000 IU of heparin unless the patient was at high risk of bleeding. This strategy is known to reduce the risk of radial artery occlusion.[14] Participating patients were randomly assigned to the study group (CD and chitosan-based pad) or control group (CD alone) in a 2:1 fashion after completion of the procedures. The random sequence was generated using a computer random number generator. The allocation numbers were kept in a locked, unreadable computer file that could be accessed only after the characteristics of an enrolled participant were entered. The study device was manufactured by a local corporation (Soyeon, Seongnam-si, Korea) and consisted of a combination of a rotatory compression pad device and chitosan-based hemostasis pads. After removal of the sheath, local compression was performed by using hemostasis pads for the study group and aseptic gauze for the control group, respectively. A compression device was then applied to deliver local pressure by moving the silicone pad for both groups. A rotatory CD alone was applied in the control group, while the CD was applied on top of the hemostasis pad for the study group.

### Outcome

The primary endpoint was time to hemostasis as a categorical variable. Hemostasis was carefully assessed every 5 minutes after applying hemostasis devices. The primary endpoint was categorized into 4 groups: ≤5 minutes, 6–10 minutes, 11–20 minutes, >20 minutes. Secondary endpoints included bleeding, hematoma, pseudoaneurysm, vessel occlusion, dissection, urgent surgical repair, vasovagal reaction, and allergic skin reaction. Bleeding events during the hospitalization were assessed according to the TIMI (Thrombolysis In Myocardial Infarction) criteria [15]. Subjective discomfort was rated using the numeric rating scales ranging from 0 to 10, with 0 representing no pain at all and 10 the worst possible pain the patient can imagine. Patients were followed up for 1 month after the index procedure.

### Statistical analysis

This study was a proof-of-concept trial. Based on our experience, we expected that 25% of the control group would achieve hemostasis within 10 minutes. It was assumed that hemostasis would be complete during the same time in 50% of patients in the study arm. Enrollment of 150 patients and randomization in a 2:1 manner was deemed to provide >85% statistical power with a significance level of 0.05. However, the sponsor faced financial instability and decided to withdraw funding during the enrollment phase of the trial after only 95 patients had participated in the study. After the interim analysis, the data safety monitoring board independently decided to stop enrolling patients because of the definitive advantages in the study arm. The actual sample size of 95 patients (59 and 36 in the study and control arms, respectively) had 71% statistical power to detect a 25% decrease in the proportion of hemostasis within 10 minutes.

The primary endpoint was compared using the chi-square test. Categorical variables were presented as numbers and percentages and were compared using the chi-square or Fisher’s exact test, as appropriate. Continuous variables were presented as the mean and standard deviation and compared using Student’s t-test. Statistical analyses were performed using R programming version 3.2.4 (The R Foundation for Statistical Computing, Vienna, Austria; http://www.R-project.org). Two-sided p<0.05 was considered statistically significant.

## Results

**Fig 1:**
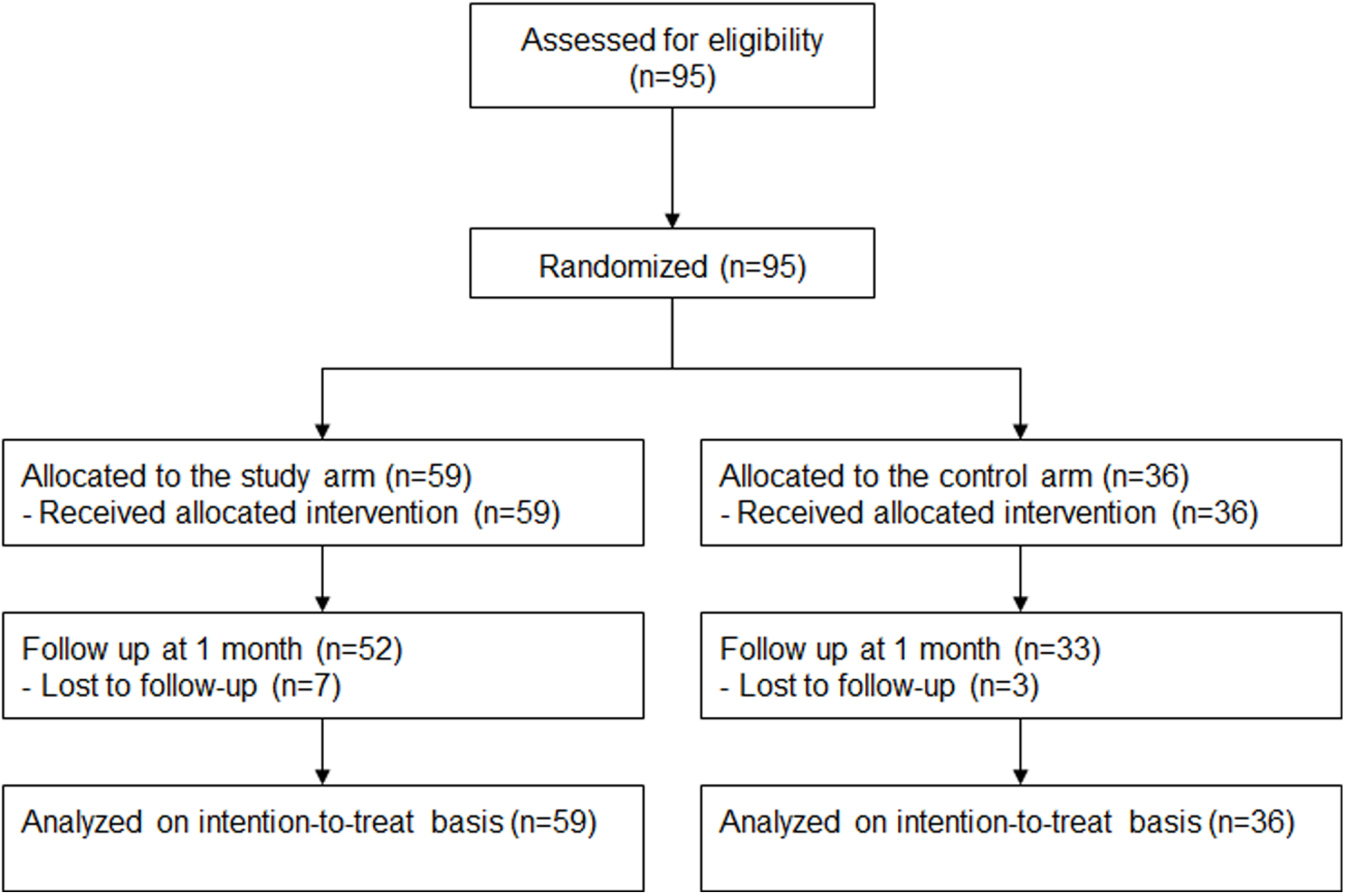
CONSORT flow chart of the study.

**Fig 2:**
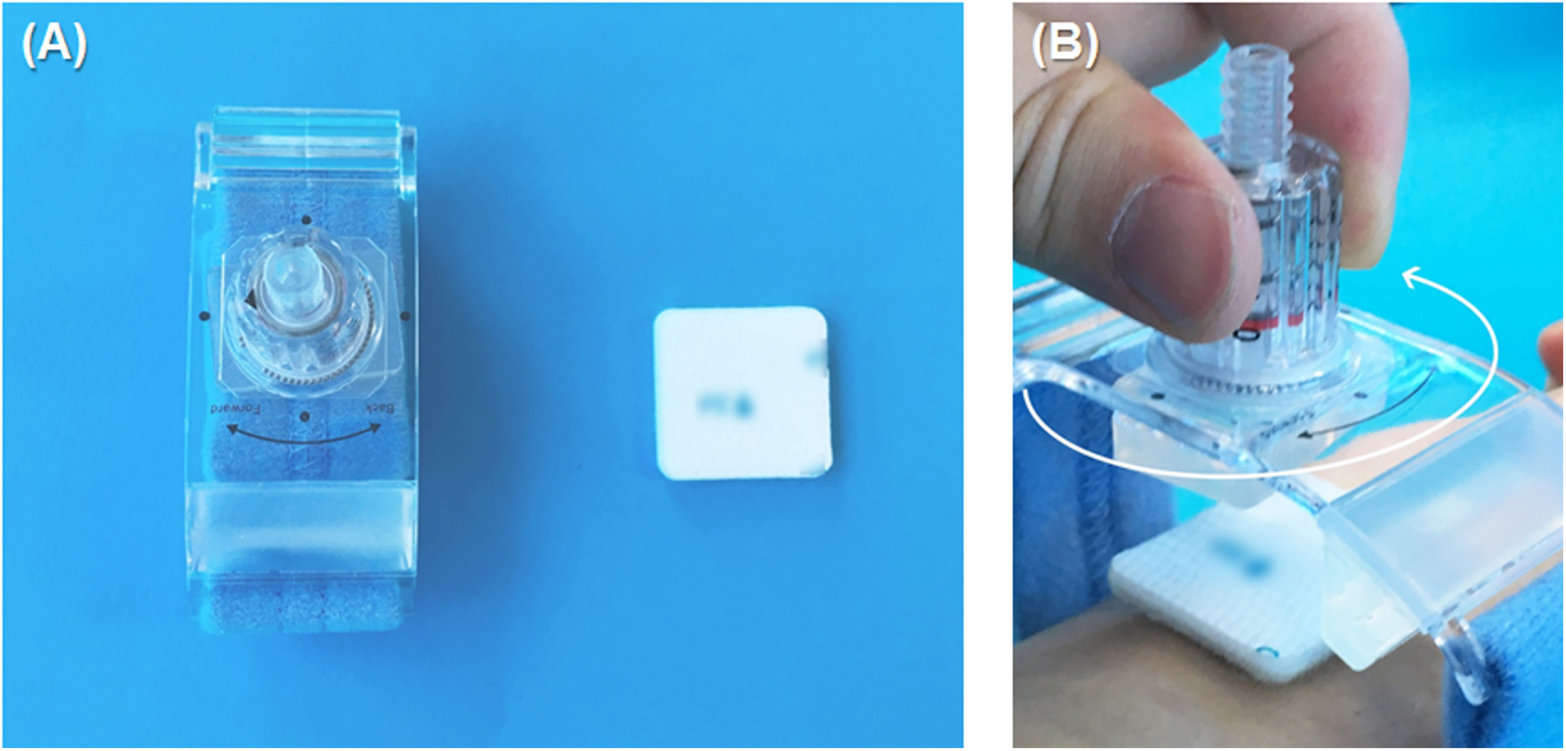
Study devices. (A) Both rotatory compression device and hemostasis pad were used for the patients in the study group. (B) After placing the hemostasis pad over the puncture site, the rotatory compression device was applied.

Between April and July 2016, 95 patients were enrolled, and all the study participants received assigned treatment (59 assigned to the study arm, and 36 to the control arm, Fig 1). Patients in the control arm received a CD after removal of the sheath, while CD was applied on top of the hemostasis pad for those in the study arm (Fig 2). There were no significant differences in baseline characteristics between the groups (Table 1). The mean age was 65 years, and 67% of the patients were men. The right radial artery was the main approach route. Five-Fr. sheaths were used in 62.1% of the subjects; 6-Fr. sheaths were used in the remaining subjects.

**Table 1:** Baseline characteristics of study patients.

	Study group	Control group
	(N = 59)	(N = 36)
Age	64.7 ± 11.9	66.0 ± 9.7
Male sex	37 (62.7%)	27 (75.0%)
Body mass index (kg/m^2^)	25.8 ± 3.4	24.1 ± 3.3
Hypertension	38 (64.4%)	24 (66.7%)
Diabetes	18 (30.5%)	13 (36.1%)
Dyslipidemia	14 (23.7%)	7 (19.4%)
Chronic renal failure	0 (0.0%)	0 (0.0%)
Smoking		
Current smoker	16 (27.1%)	5 (13.9%)
Former smoker	8 (13.6%)	10 (27.8%)
Never smoker	35 (59.3%)	21 (58.3%)
Heavy drinker	4 (6.8%)	2 (5.6%)
Laboratory tests		
Hemoglobin (g/dL)	13.5 ± 1.6	13.7 ± 1.7
Platelet count (/μL)	256.5 ± 84.4	223.6 ± 66.3
Total bilirubin (mg/dL)	0.8 ± 1.5	0.6 ± 0.3
AST (mg/dL)	30 ± 25	30 ± 22
ALT (mg/dL)	25 ± 18	38 ± 64
PT	1.0 ± 0.1	1.0 ± 0.1
aPTT	36.0 ± 5.9	36.5 ± 7.0
Procedure		
Coronary angiography	58 (98.3%)	34 (94.4%)
Percutaneous coronary intervention	1 (1.7%)	2 (5.6%)
Clinical indication		
Stable angina	28 (47.5%)	17 (47.2%)
Unstable angina	5 (8.5%)	9 (25.0%)
Non ST-elevation myocardial infarction	1 (1.7%)	2 (5.6%)
Variant angina	7 (11.9%)	5 (13.9%)
Chest pain of non-cardiac origin	6 (0.2%)	2 (5.6%)
Non-coronary artery disease	12 (20.3%)	1 (2.8%)
Previous transradial procedures	5 (8.2%)	3 (9.1%)
Vascular approach		
Left	3 (5.1%)	2 (5.6%)
Right	56 (94.9%)	34 (94.4%)
Sheath size		
4 Fr	0 (0.0%)	1 (2.8%)
5 Fr	40 (67.8%)	19 (52.8%)
6 Fr	19 (32.2%)	16 (44.4%)
7 Fr	0 (0.0%)	0 (0.0%)
Medications		
Use of heparin	3 (5.1%)	4 (11.1%)
Aspirin	44 (74.6%)	32 (88.9%)
Clopidogrel	39 (66.1%)	30 (83.3%)
Prasugrel/ticagrelor	1 (1.7%)	1 (2.8%)
Vital status		
Systolic blood pressure (mmHg)	131.7 ± 19.2	137.0 ± 16.9
Diastolic blood pressure (mmHg)	78.0 ± 13.3	82.4 ± 10.5
Heart rate (/min)	69.8 ± 11.3	69.3 ± 12.3

Abbreviations: AST, aspartate transaminase; ALT, alanine transaminase; PT, prothrombin time; aPTT, activated partial thromboplastin time

**Fig 3:**
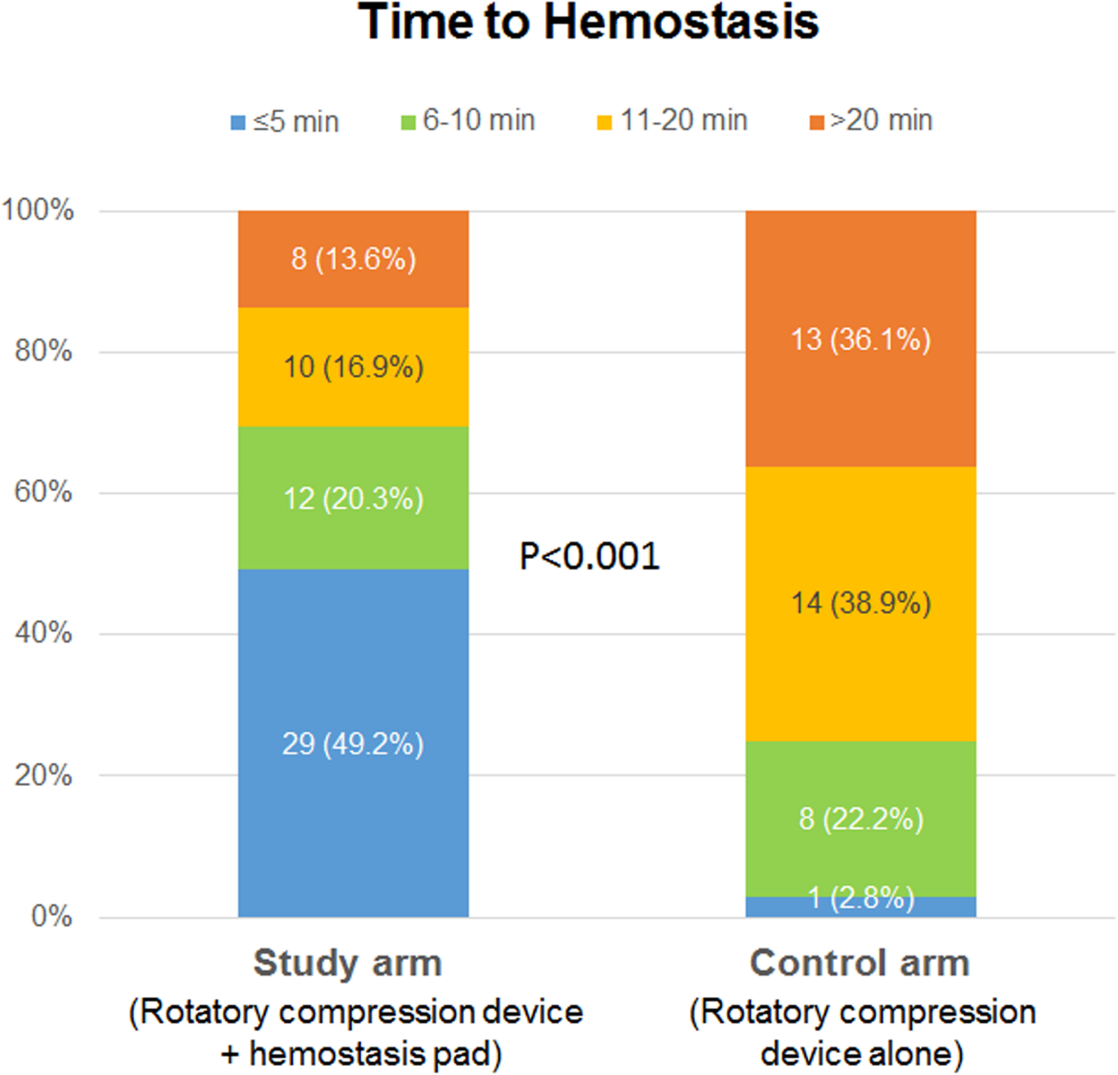
The primary endpoint, time to hemostasis of the study and control groups.

Fig 3 shows the results of the primary endpoint. The proportion of patients who reached hemostasis within 5 minutes, 6–10 minutes, 11–20 minutes, and > 20 minutes significantly differed between the groups (p<0.001). While 69% of the patients in the study group achieved hemostasis within 10 minutes, it took more than 11 minutes to achieve hemostasis in 75% of the patients in the control group.

**Fig 4:**
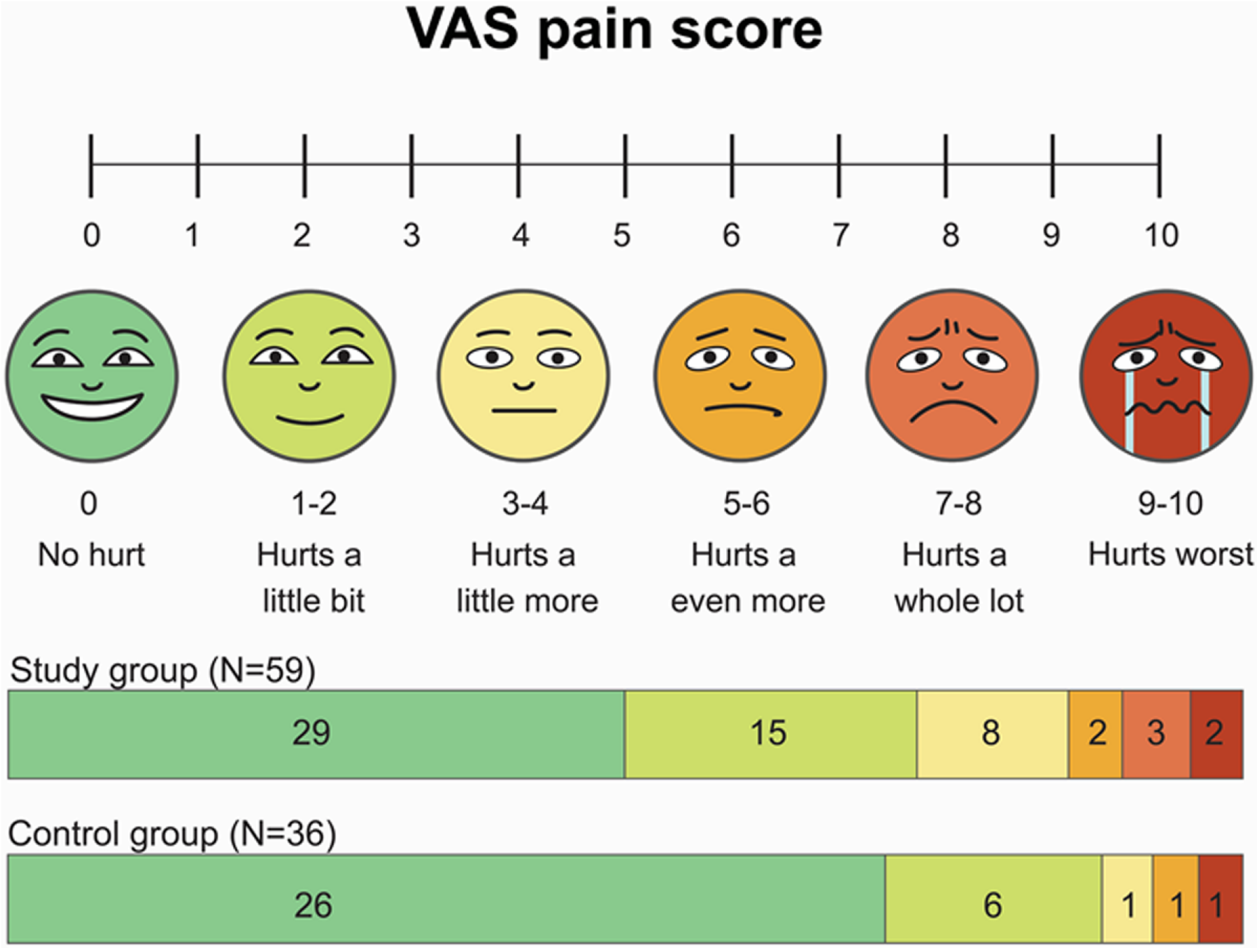
Subjective discomfort assessed by visual analogue scale.

Both groups showed low rates of vascular complications (Table 2). There were no TIMI major bleedings, and 1 patient from each group developed hematoma. Skin rash occurred in 2 patients in the study group. Subjective discomfort assessed by the numeric rating scales tended to be greater in the study group but did not differ significantly (p = 0.197) (Fig 4).

**Table 2:** Procedural complications.

	Study group	Control group
	(N = 59)	(N = 36)
Bleeding	5 (8.5%)	3 (8.3%)
TIMI major bleeding	0 (0.0%)	0 (0.0%)
TIMI minor bleeding	0 (0.0%)	0 (0.0%)
Oozing	5 (8.5%)	3 (8.3%)
Hematoma	1 (1.7%)	1 (2.8%)
Vessel occlusion	0 (0.0%)	0 (0.0%)
Urgent vascular surgery	0 (0.0%)	0 (0.0%)
Rash	2 (3.4%)	0 (0.0%)

## Discussion

Arterial access management is a key process during percutaneous cardiovascular procedures.[16] Rotatory CD and the chitosan-based hemostasis pad are both widely used hemostasis strategies in clinical practice after radial artery access. This prospective randomized controlled trial demonstrated that the combination of the two hemostasis strategies was safe and superior to CD alone in reducing the time to hemostasis. In addition, this strategy was not associated with an increased risk of vascular complications.

Studies have reported lower rates of bleeding and access-site vascular complications with the transradial approach.[17–20] In addition, radial access has been shown to reduce the risks of mortality and bleeding compared to the femoral approach in patients with acute coronary syndrome.[21] Current guidelines prefer radial access over femoral access if performed by experienced operators.[22, 23] However, the transradial approach is still not free from access site complications although they are low [3]. Major bleeding is an independent predictive factor of adverse clinical outcomes regardless of the access site.[24] In addition, hemostasis of the access site is one of the fundamental aspects of coronary procedures. This study demonstrated improved hemostasis efficacy with no additional complications when hemostasis pads were used on top of CD. A previous study also suggested that a reduced hemostatic compression time is associated with a lower risk of vascular complications such as radial artery occlusion.[25, 26]

The only concern raised in this study was the possible increase in allergic reactions. Chitosan is produced by deacetylation of chitin, which is extracted from the shells of shrimps, lobsters, and beetles. The positively charged chitosan molecules attract the negatively charged blood cells and platelets, thus promoting blood clotting. The safety of chitosan-based hemostasis pads has been shown in previous studies.[11, 27] In this study, the frequency of allergic reactions was low, and no patients developed severe allergic reactions.

This study has several limitations. First, this study was stopped prematurely before enrollment of the planned number of patients. However, the benefit shown in this study group was definite despite the small sample size. Second, we hypothesized that a reduction in time to hemostasis may lead to a decrease of vascular complications such as radial artery occlusion. However, although the difference in the efficacy endpoint was significant, the occurrence of safety endpoints was too low to show any difference. Therefore, a 30-day assessment of the vascular access site with ultrasound would have improved the quality of this study. Future studies with ultrasound follow-up are needed to evaluate the safety profile of this novel hemostasis approach. Third, percutaneous intervention was performed in a small proportion of the study patients.

In conclusion, this study suggests that the addition of chitosan-based pads on top of rotatory CD may be an effective and safe strategy for puncture site hemostasis after radial artery access.

## Supporting information

S1 File Consort check list. Consort 2010 check list.doc.
(DOC)

S2 File Study protocol(English version). 8_Protocol_ezclot(English)_20170402.docx.
(DOCX)

S3 File Study protocol(Korean version). 9_Protocol_ezclot(Korean).docx.
(DOCX)
