# Supplementary material for: Hemostasis pad combined with compression device after transradial coronary procedures: A randomized controlled trial
Source: PLoS One. 2017 Jul 24;12(7):e0181099. doi: 10.1371/journal.pone.0181099 (PMC5524363; doi:10.1371/journal.pone.0181099)
Supplement: S2 File — 8_Protocol_ezclot(English)_20170402.docx. (DOCX) [file pone.0181099.s002.docx]

**Study Protocol**

**1. Title**

Hemostatsis pad using chitosan after invasive coronary or peripheral procedures

**2. Institution and address**

Seoul National Univiersity Bundang Hospital

39222, 82, Gumi-ro 173 beon-gil Bundang-gu Seongnam-si Gyeonggi-do, Republic of Korea

**3. Research request institution**This study is an investigator initiated trial.

**4. Sponsor legal registered address**

Seongnam industry promotion agency (<https://www.SNVENTURE.NET)>

13558, 7^th^ floor, 8, Seongnam-daero 331beon-gil, Bundang-gu, Seongnam-si, Gyeonggi-do, Republic of Korea

**5. Expected research duration**

From the approval of the study protocol by the institutional review board to December 2016

**6. Subjects of research**

Patients who underwent coronary angiography for coronary artery disease or peripheral artery disease

**7. Background and object**

**1) Background**

The prevalence of cardiovascular disease is increasing and it is a major cause of death. Therefore, transarterial examinations such as coronary angiography and intervention has become important tools of diagnosis and treatment for cardiovascular disease. The procedures are usually performed through radial or femoral artery. And hemostasis after procedure is an important issue. Direct external compression on punctured site takes long time to control bleeding can cause discomfort to patients.^1^ Also, the success rate of hemostasis with this method was low. To overcome this problem, compression devices have been developed and used in the clinical field.^2-4^ HemCon^®^ pad (HemCon Medical Technologies, Inc., Portland OR, USA) is a world-widely applied hemostatic device, which is based on synthetic chitosan made from chitin.^5^ Chitin is obtained easily from shellfish and insects, and the mechanism of action is based on ionic interaction between the positively loaded chitosan and negatively loaded red blood cells and platelets. Recently, a domestic company developed a chitosan-based pad, named ezClot^®^ (Soyeon, Seongnam-si, Korea) and it was manufactured similar with HemoCon^®^ pad. The ezClot^®^ was approved by Korean Food and Drug Administration. Therefore, our study evaluates the safety and hemostatic effect of ezClot^®^.


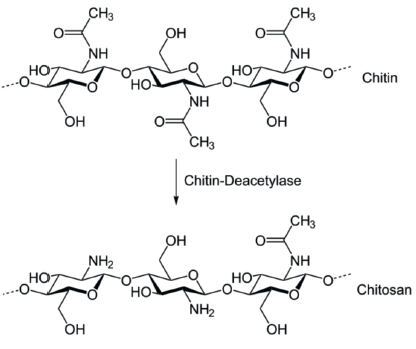


**2) Hypothesis and object**

For radial subgroup, we hypothesized that the combination of compression device (CD) with chitosan-based pads would improve hemostasis efficacy and safety compared with control devices after coronary angiography or interventions.

For femoral subgroup, we hypothesized the ezClot pads would show similar hemostasis efficacy compared to BloodSTOP^®^ ix pads.

**8. Study materials and devices**

- Study device : ezClot^®^ (Chitosan-based pad)
- Control device :

① Femoral puncture subgroup: BloodSTOP^®^ ix
(soluble, oxidized–etherified regenerated cellulose)

② Radial puncture subgroup: manual compression with compression device

**9. Inclusion/exclusion criteria**

**i) Inclusion criteria**

patients aged ≥18 years

Those with elective or urgent coronary angiography or interventions

**ii) Exclusion criteria**

congenital or acquired bleeding tendency

thrombocytopenia (platelet count < 50,000/uL)

patients with hypersensitivity for shellfish

**iii) Sample size**

This trial is a proof-of-concept trial. To prove the hypothesis that the study arm has better or equal hemostasis efficacy than the control arm, this study will enroll a total of 315 patients.

- Femoral puncture subgroup: Study arm 100 patients + control arm 50 patients

- Radial puncture subgroup: Study arm 100 patients + control arm 50 patients

Number considered the rate of drop out (5%): Study arm 210 patients,

Control arm 105 patient

🡪 total 315 patients

**iv) Patient recruitment**

This study will recruit the patients who were admitted to perform invasive procedure for cardiovascular diseases. If the patient is suitable for this study, the researcher will explain the purpose of study and provide written consent to the patients and patients’ guardians. The principal researcher and research institution do not exclude the patients distinguishing from race, economic state. If the patients are suitable for inclusion criteria, we help them to participate in the research.

**10. Research Methods**

**i) Detailed descriptions of the protocols**

Before coronary or peripheral angiography, the potential candidates will be screened. Participant will give their written informed consents before the procedures. Decision of the participation into the study will be decided immediately after the procedures. Study patients will be stratified into the radial or femoral subgroups.

Study patients will be randomly assigned to the study or control groups in a 2:1 fashion after completion of the procedures. Random sequence will be generated using a computer random number generator. The allocation numbers will be kept in a locked unreadable computer file, and could be accessed only after the characteristics of an enrolled participant have been entered.

The primary endpoint is time to hemostasis. The Secondary endpoints include bleeding, hematoma, pseudoaneurysm, vessel occlusion, dissection, urgent surgical repair, vasovagal reaction, and allergic skin reaction.

**ii) Randomization method**

control arm : Femoral puncture group : BloodSTOP^®^ ix

Radial puncture group : manual compression with compression device

We randomized with world wide web <http://stattrek.com/Tables/Rsndom.aspx> (study device = 1/ control = 0). Randomization was followed simple randomization.

**iii) Devices utilizing method**

Hemostasis is performed by compression to all patients. However, the selection of devices is randomized. The manual of study device, ezClot^®^ is as follows:

- 1.
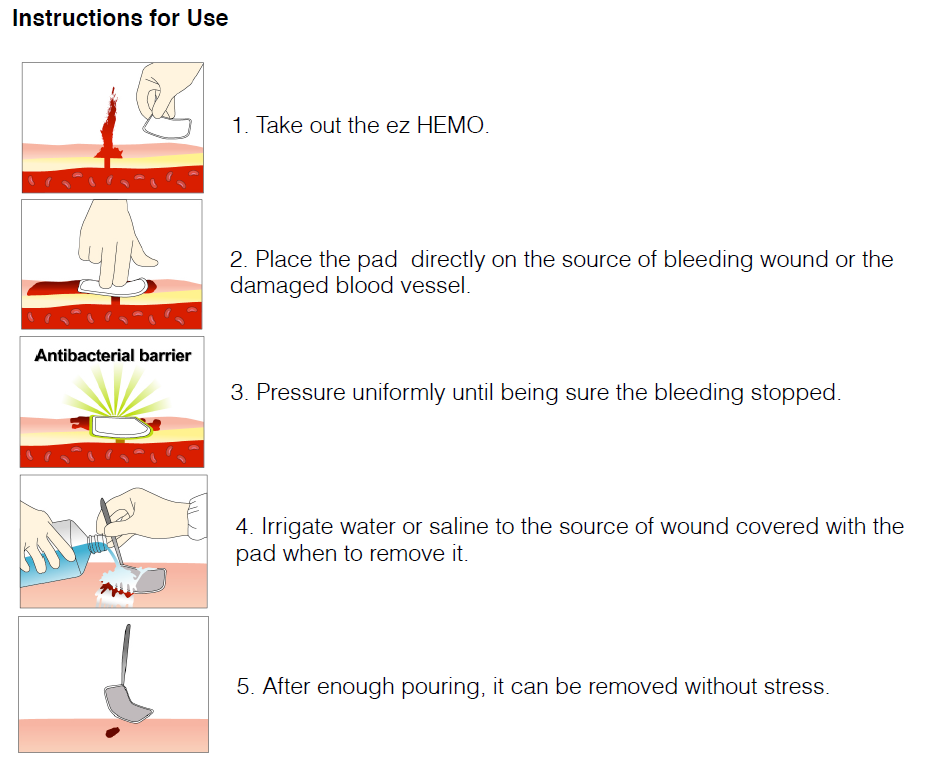
Pad type


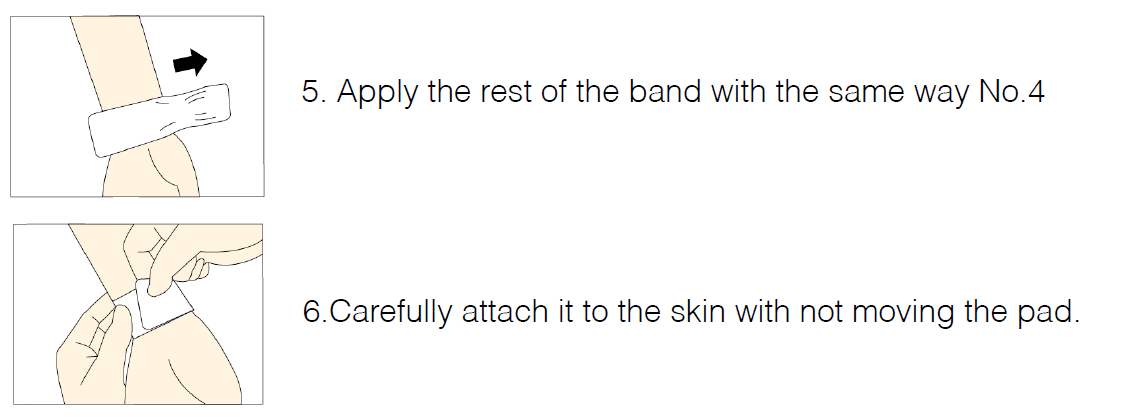

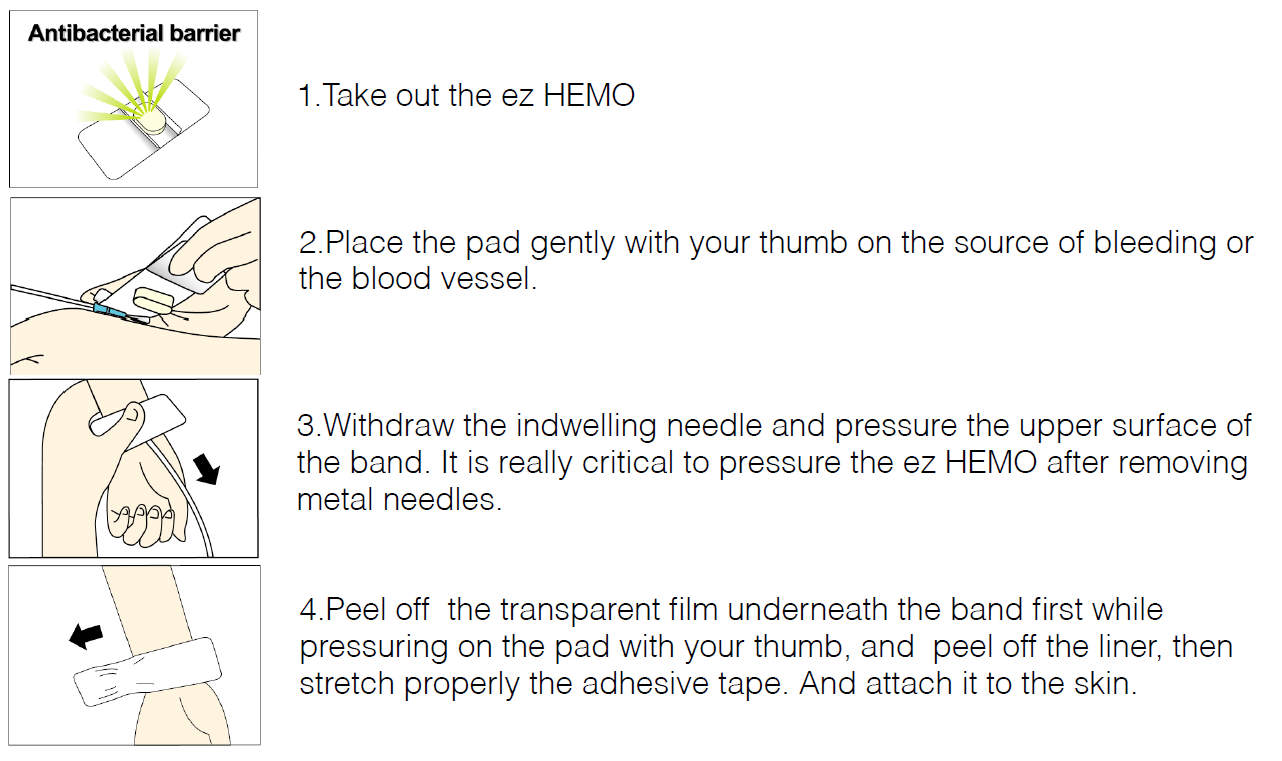
Bandage type

**iv) Study endpoints**

- Primary end point : time to hemostasis

- Secondary end point : vascular complication after hemostasis

- Bleeding (TIMI definition: major, minor, minmal)

- Hematoma

- Retroperitoneal hematoma

- Pseudoaneurysm

- Vessel occlusion

- Dissection

- Surgical repair

- Vasovagal reaction

**i) Schematic protocol**

| Variables | Baseline | Follow Up | |
| --- | --- | --- | --- |
|  |  | Post-Procedure (1day) | 30 days(±2w) |
| History taking | O | O | O |
| Physical examination | O | O | O |
| CBC | O | O | O |
| Time to hemostasis | O | X | X |
| Hemorrhage, hematoma, pseudoaneurysm | O | O | O |
| Distal flow | O | O | O |
| Subjective discomfort | X | O | X |

**11 References**

1. Cong X, Huang Z, Wu J, Wang J, Wen F, Fang L, Fan M and Liang C. Randomized Comparison of 3 Hemostasis Techniques After Transradial Coronary Intervention. J Cardiovasc Nurs. 2015.

2. Trabattoni D, Montorsi P, Fabbiocchi F, Lualdi A, Gatto P and Bartorelli AL. A new kaolin-based haemostatic bandage compared with manual compression for bleeding control after percutaneous coronary procedures. Eur Radiol. 2011;21:1687-91.

3. Trabattoni D, Gatto P and Bartorelli AL. A new kaolin-based hemostatic bandage use after coronary diagnostic and interventional procedures. Int J Cardiol. 2012;156:53-4.

4. Politi L, Aprile A, Paganelli C, Amato A, Zoccai GB, Sgura F, Monopoli D, Rossi R, Modena MG and Sangiorgi GM. Randomized clinical trial on short-time compression with Kaolin-filled pad: a new strategy to avoid early bleeding and subacute radial artery occlusion after percutaneous coronary intervention. J Interv Cardiol. 2011;24:65-72.

5. Arbel J, Rozenbaum E, Reges O, Neuman Y, Levi A, Erel J, Haskia AR, Caneti M, Sherf M and Mosseri M. USage of chitosan for Femoral (USF) haemostasis after percutaneous procedures: a comparative open label study. EuroIntervention. 2011;6:1104-9.
