# Supplementary material for: Hemostasis pad combined with compression device after transradial coronary procedures: A randomized controlled trial
Source: PLoS One. 2017 Jul 24;12(7):e0181099. doi: 10.1371/journal.pone.0181099 (PMC5524363; doi:10.1371/journal.pone.0181099)
Supplement: S3 File — 9_Protocol_ezclot(Korean).docx. (DOCX) [file pone.0181099.s003.docx]

**(국문) 관혈적 중재술 후 키토산 지혈패드의 지혈 효과 비교 연구**

**(영문) Hemostatsis pad using chitosan after invasive coronary or peripheral procedures**

**Version No: 1.0**

**책임연구자 소속: 심장혈관센터**

**책임연구자 이름: 채인호**

**연구 개요**

| 연구제목 | (국문) 관혈적 중재술 후 키토산 지혈패드의 지혈 효과 비교 연구 |
| --- | --- |
|  | (영문) Hemostatsis pad using chitosan after invasive coronary or peripheral procedures |
| 책임연구자 | 내과 채인호 교수 |
| 연구비 지원기관 | 성남산업진흥재단  (우.13558)경기도 성남시 분당구 성남대로 331번길 8, 7층(정자동) |

| 연구 목적 | 심혈관계 질환으로 관혈적 중재술 후 출혈을 억제하기 위한 목적으로 키토산 지혈패드를 사용하여 지혈까지 걸리는 시간과 합병증의 발생, 환자의 만족도를 평가하는 것을 목적으로 한다. |
| --- | --- |
| 연구 설계 | Prospective randomized trial |
| 연구 기간 | IRB승인일 ~ 2016년 12월 |
| 연구 대상 | 관상동맥질환 또는 말초동맥 질환으로 관혈적 혈관 조영술을 시행하는 환자 |
| 연구 대상자 수 | 총 315명 (중도탈락율 5%를 가정) |
| 취약한 연구대상자 | 해당 없음. |
| 시험약/의료기기 | ezClot (키토산을 이용한 지혈패드) |
| 의료기기 관리자 | 심혈관 조영실에서 주로 중재술이 끝남과 동시에 즉시, 출혈을 억제하기 위한 목적으로 지혈패드를 적용, 지혈이 완료될 때까지 심혈관계 중환자실에서 합병증 등을 관찰해야 하므로 3층 심혈관 조영실에 상주중인 공동연구자 **강시혁**을 의료기기 관리자로 지정합니다. |
| 용법 및 용량 | 압박지혈술 시 혈관 천자 부위에 적용한다. |
| 연구 방법 | 관상동맥조영술 또는 말초혈관조영술을 위해 입원한 환자가 screening 대상이 된다. 시술 전 사전에 환자의 동의를 획득한 후, 검사가 종료되는 시점에서 본 연구에 참여 여부를 결정한다. 연구대상자는 femoral puncture group과 radial puncture group으로 층화하여 enroll하게 된다.  자발적 동의 하에 연구에 참여한 환자들은 시험기기군 또는 대조기기군으로 2:1의 비율로 randomization 된다. 시험기기군에 배정된 환자는 ezClot를 이용하여, 대조기기군에 배정된 환자는 대퇴동맥 천자군인 경우 BloodSTOP ix로 요골동맥 천자군인 경우 압박지혈술로 지혈하게 된다. |
| 주요 선정기준 | 1. 만 18세 이상의 성인  2. 심혈관계 질환으로 대퇴동맥 또는 요골동맥을 이용한 침습적 시술을 받는 환자 |
| 선정 제외기준 | 1. 유전적 또는 후천적인 출혈 경향을 가지고 있는 환자  2. platelet count <50,000/ μL  3. 갑각류에 과민 반응을 가지고 있는 환자 |
| 유효성 평가 | 유효성 평가기준 :   \|  \| 대 퇴 동 맥 천 자 군 \| 요 골 동 맥 천 자 군 \| \| --- \| --- \| --- \| \| 1차 유효성  평가기준 \| 지혈까지의 시간  (time to hemostasis, min) \| 혈관합병증 발생 빈도  1)반상출혈 (ecchymosis)  2)출혈 (woozing)  3)혈종 (hematoma)  4)가동맥류(pseudoaneurysm) \| \| 2차 유효성  평가기준 \| 혈관합병증 발생 빈도  1)반상출혈 (ecchymosis)  2)출혈 (woozing)  3)혈종 (hematoma)  4)가동맥류(pseudoaneurysm)  주관적 불편감 (visual analogue scale) \| 요골동맥폐색 (radial artery occlusion)  주관적 불편감 (visual analogue scale) \|   유효성 통계분석방법 :  지혈까지의 시간, 주관적 불편감: Student's t-test  혈관합병증 발생 빈도, 요골동맥 폐색: chi square test |
| 안전성 평가 | 안전성 평가기준 :  - 알레르기 등 과민반응  - 접촉성 피부염  - 예기치 못한 사망 등 기존의 질환으로 설명하기 어려운 임상 경과  안전성 통계분석방법 :  Chi square test 또는 Fisher's exact test |
| 검사/방문일정 | \| 변 수 명 \| 스크리닝 \| 시험기간 \| \| \| --- \| --- \| --- \| --- \| \| 시술 당일 \| 방문 1  시술 후 1일 \| 방문 2  시술 후 4주 \| \| 문 진 \| O \| O \| O \| \| 신 체 검 진 \| O \| O \| O \| \| CBC (총혈구검사) \| O \| O \| O \| \| 지혈까지의 시간 \| O \| X \| X \| \| 출혈, 혈종, 가동맥류 발생 여부 \| O \| O \| O \| \| 원위부 혈류 \| O \| O \| O \| \| 설문지를 통한 불편도 평가 \| X \| O \| X \| |
| 통계적 분석방법 | Primary endpoint는 Student’s t-test를 통해, secondary endpoints들은 chi square test를 통해 검정한다. |
| 기대효과 및  예상결과 | 본 연구의 시험기기인 ezClot은 국내자본에 의해 개발된 지혈기구로서, 본 기기의 효용성과 안정성을 증명하는 것이 본 연구의 목적이다. 또한 본 연구는 관상동맥조영술과 말초혈관조영술을 시행한 환자들의 출혈 위험을 면밀하게 평가하도록 설계되어 있어 검사 후 출혈과 지혈에 대한 소중한 임상정보를 제공할 것으로 기대된다. |

**연구계획서**

1. **연구 제목**

(국문) 관혈적 중재술 후 키토산 지혈패드의 지혈 효과 비교 연구

(영문) Hemostatsis pad using chitosan after invasive coronary or peripheral procedures

1. **연구의 실시기관 명칭 및 주소**

분당서울대학교병원, 464-707, 경기도 성남시 분당구 구미로 166

1. **연구 의뢰기관**연구자 주도 임상시험임. 해당 없음.
2. **연구비 지원기관 명칭 및 주소**

성남산업진흥재단 SNVENTURE.NET

(우.13558)경기도 성남시 분당구 성남대로 331번길 8, 7층(정자동)

1. **예상연구기간**

IRB 승인일 ~ 2016년 12월

1. **연구 대상 질환**

관상동맥질환 또는 말초동맥 질환으로 관혈적 혈관 조영술을 시행하는 환자

1. **연구의 배경 및 목적**

**1) 연구 배경**

심혈관계 질환은 세계적으로 유병율이 높고 주요 사망 원인 중 하나로 손꼽힌다. 이와 같은 심혈관계 질환에 있어서 관혈적 검사와 중재시술은 중요한 진단/치료의 과정으로 자리잡게 되었는데, 이와 같은 관혈적 시술은 요골동맥이나 대퇴동맥을 통해 이루어지게 된다. 이와 같은 동맥을 통한 시술 후 지혈이 중요한 문제가 된다. 전통적으로 사용하던 방법은 검사자가 직접 압박지혈을 가거나 압박 도구를 이용해 장시간 압박지혈하는 방법인데, 이러한 압박지혈법은 환자에게 큰 불편을 초래하고 시간이 오래 걸리며 실패율이 비교적 높다는 것이 단점으로 꼽힌다.^1^

이와 같은 어려움을 극복하기 위해 지혈을 돕는 지혈패드가 개발되어 현재 임상에서 널리 사용되고 있다.^2-4^ HemCon® pad (HemCon Medical Technologies, Inc., Portland OR, USA) 가 현재 미국을 포함한 여러나라에서 가장 많이 쓰고 있는데, 이는 키틴을 화학적으로 조작하여 합성한 키토산을 이용한 지혈방법이다.^5^ 키틴은 갑각류와 곤충에서 쉽게 구할 수 있는데, 양전하를 띤 키토산이 음전하를 띠고 있는 혈구세포 및 혈소판과 만나 지혈을 촉진하게 된다. 최근 국내 업체에서도 키토산을 이용한 지혈도구를 제작하여 식약처 승인을 받았다. ezClot이라고 명명된 이 지혈 패드는 기존의 HemoCon pad와 거의 동일한 방법으로 제작되었다. 이에 본 연구에서는 국내 업체에서 개발한 ezClot의 지혈효과를 평가하는 것을 목적으로 한다.


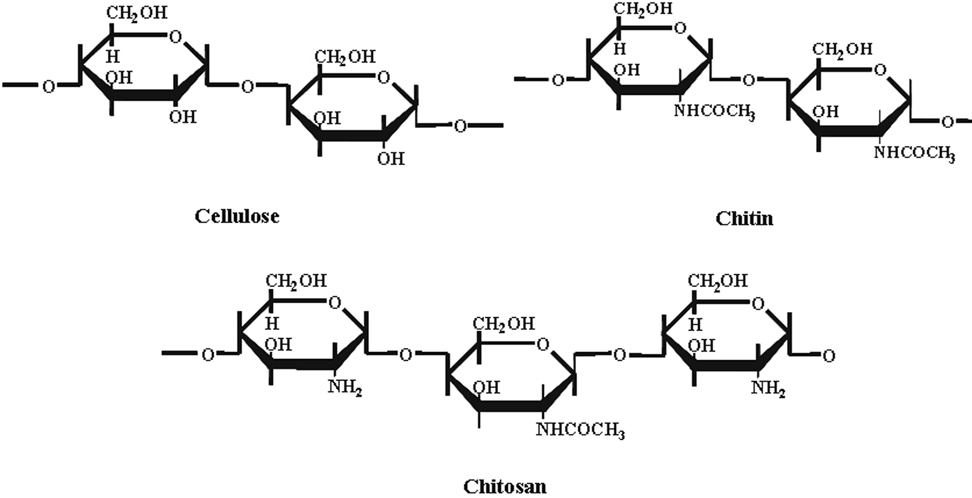
그림 1. 키토산 구조와 합성과정


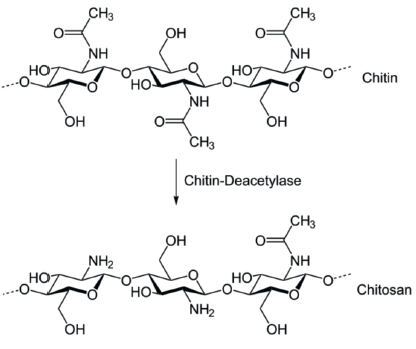


**2) 연구 가설 및 목적**

본 연구는 관혈적 심혈관계 시술을 받은 환자에서 ezClot 패드의 효과와 안전성을 평가하는 것을 목적으로 한다. 본 연구에서는 ezClot을 기존에 사용되던 HemCon 패드와 비교하여 비슷한 지혈효과와 안전성을 가지는지 확인한다.

1. **임상시험용 의약품 및 의료기기 코드명(또는 주성분의 일반명), 원료약품의 분량, 제형 등(대조약 포함)**

- 시험기기 : ezClot (키토산을 이용한 지혈패드)
- 대조기기 :

① 대퇴동맥 천자군: BloodSTOP ix (셀룰로오스 지혈 패드)

② 요골동맥 천자군: 압박지혈술

1. **연구대상자의 선정 기준, 제외기준, 목표한 대상자 수 및 산출 근거**
2. **선정기준**
   - 1. 만 18세 이상의 성인
     2. 심혈관계 질환으로 대퇴동맥 또는 요골동맥을 이용한 침습적 시술을 받는 환자
3. **제외기준**
   - 1. 유전적 또는 후천적인 출혈 경향을 가지고 있는 환자
     2. platelet count <50,000/ μL
     3. 갑각류에 과민 반응을 가지고 있는 환자
4. **목표한 대상자 수 및 산출 근거**

본 연구는 proof-of-concept trial로 다음과 같은 피험자를 등록하는 것을 목표로 한다.

- 대퇴동맥 천자군 : 시험군 100명 + 대조군 50명
- 요골동맥 천자군 : 시험군 100명 + 대조군 50명

탈락율(5%)를 감안한 피험자 수 : 시험군 210명, 대조군 105명, 총 315명

1. **연구 대상자 모집 계획**

본 연구는 심혈관계 질환에 대한 침습적 시술을 위해 입원한 환자를 대상으로 한다. 적절한 연구 대상이라고 판단되는 경우, 시술 전 환자/보호자에게 연구의 목적에 대해 설명하고 동의를 취득한다. 본 연구의 책임연구자 및 의뢰기관은 인종이나 사회경제적 상태에만 근거해서 이 연구에 참여할 가능성이 있는 환자를 배제시키지 않을 것이다. 이 연구의 선정기준에 합당하다면, 가능한 환자들이 이 연구에 참여할 수 있도록 모든 노력을 다할 것이며 본 기관에서 치료받는 서맥 환자의 전체를 대표할 수 있도록 환자들에게 연구의 목적을 주지시킬 것이다.

1. **연구 방법**
2. **구체적인 연구방법**

관상동맥조영술 또는 말초혈관조영술을 위해 입원한 환자가 screening 대상이 된다. 시술 전 사전에 환자의 동의를 획득한 후, 검사가 종료되는 시점에서 본 연구에 참여 여부를 결정한다. 연구대상자는 femoral puncture group과 radial puncture group으로 층화하여 enroll하게 된다.

자발적 동의 하에 연구에 참여한 환자들은 시험기기군 또는 대조기기군으로 2:1의 비율로 randomization 된다. 시험기기군에 배정된 환자는 ezClot를 이용하여, 대조기기군에 배정된 환자는 대퇴동맥 천자군인 경우 BloodSTOP ix로 요골동맥 천자군인 경우 압박지혈술로 지혈하게 된다.

Primary endpoint는 time to hemostasis로 심뇌혈관 조영실에서 지혈이 완료될 때까지의 시간으로 정의한다. Secondary endpoint는 퇴원 전 입원 기간 동안 발생한 합병증을 포함한다.

1. **비교군 설정 및 무작위 배정 방법**

비교군 : ① 대퇴동맥 천자군: BloodSTOP ix (셀룰로오스 지혈 패드)

② 요골동맥 천자군: 압박지혈술

인터넷 웹 사이트 http://stattrek.com/Tables/Random.aspx를 통해 무작위 배정표를 작성한다 (LV pacing = 1 / RV pacing = 0). 무작위배정은 simple randomization 방법을 따른다. 무작위 배정은 central randomization 되어 배정군 정보를 연구자가 사전에 알 수 없도록 하며 (allocation concealment),하여 random이 결정된 직후 website를 통해 배정군 정보를 확인하도록 한다.

1. **시험약 투여∙사용량, 투여∙사용 방법, 병용 요법, 대조약 사용시 그 선택사유**

시험군 과 대조군 모두 압박지혈술을 이용한 방법으로 지혈을 하게 되며, 이 때 사용하는 지혈기구만 random하여 배정하게 된다. 시험기기인 ezClot의 사용법은 다음과 같다.

- 1.
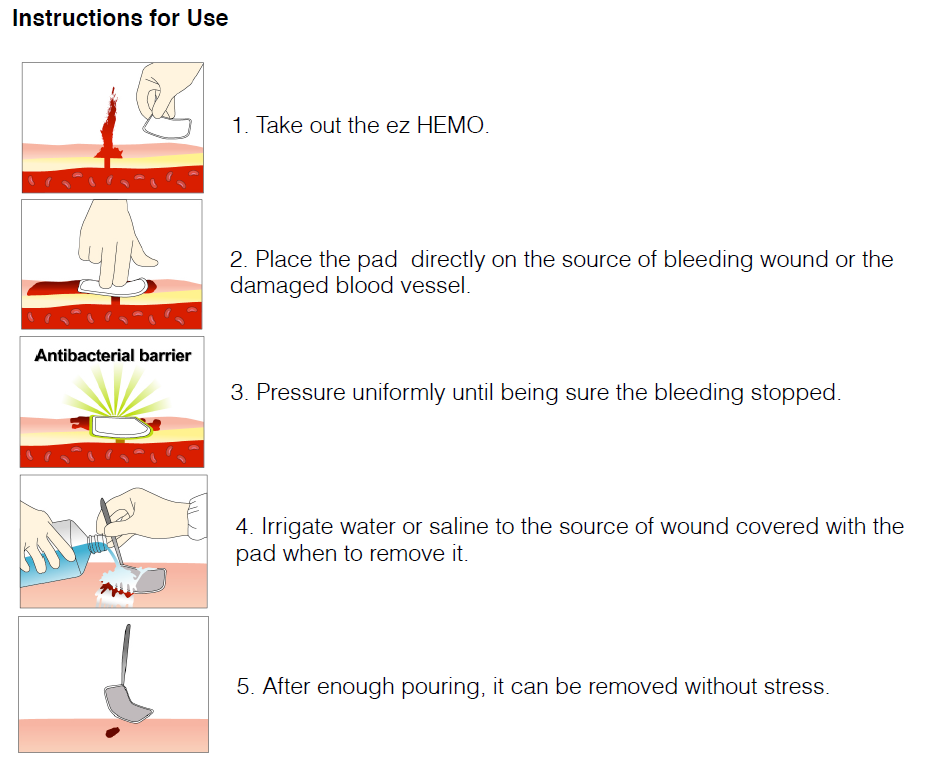
Pad type
  2. Bandage type


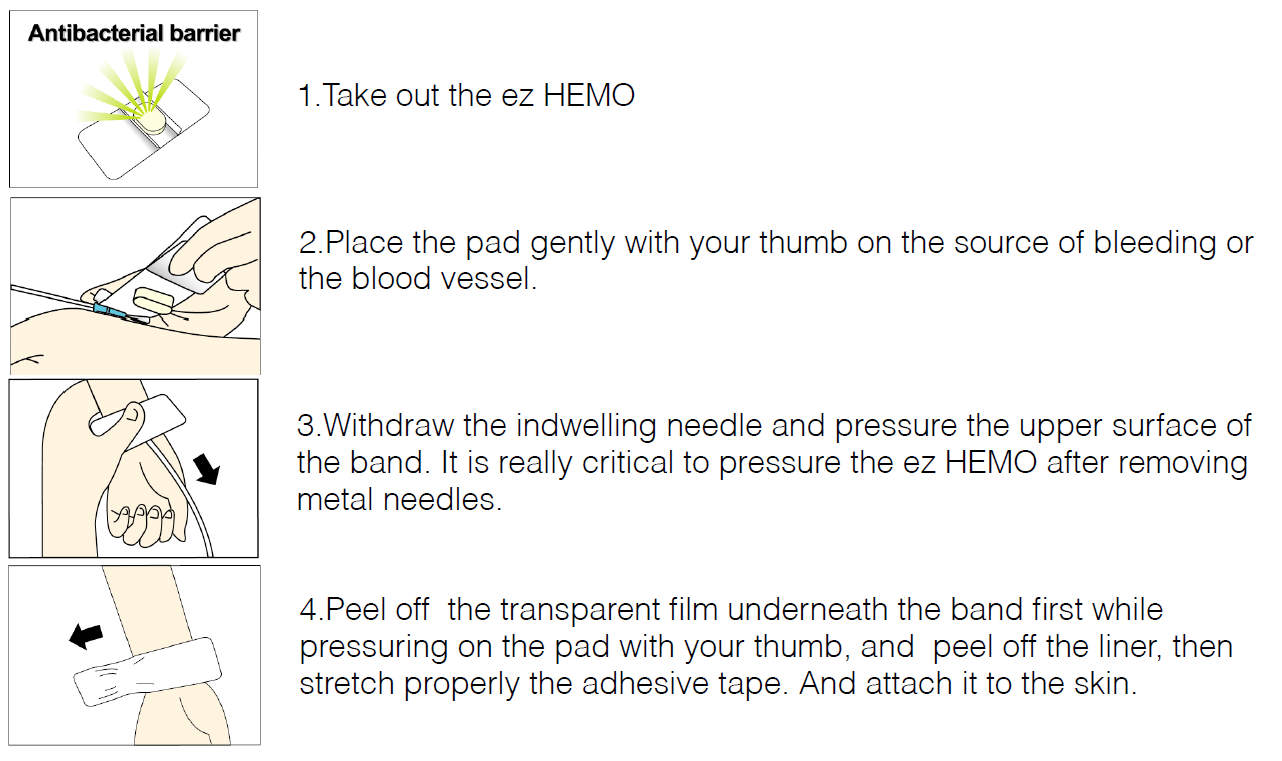


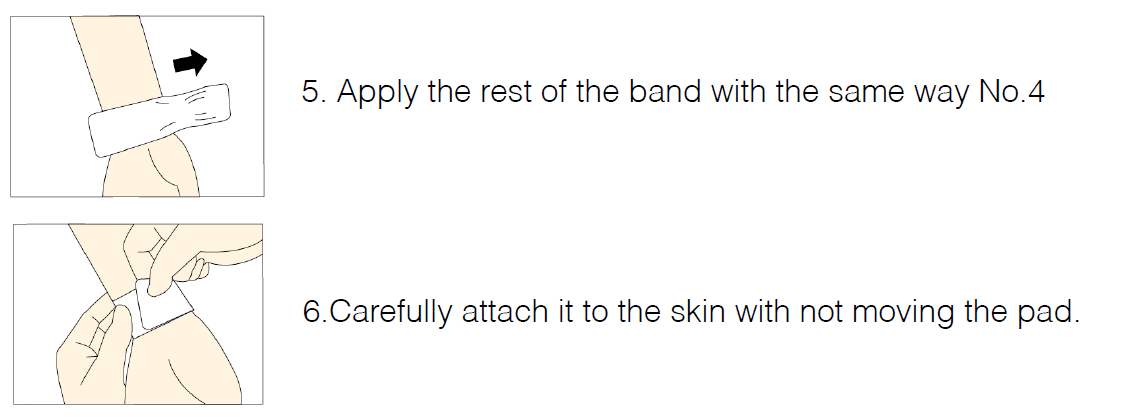


1. **관찰항목, 임상검사항목 및 관찰검사방법**

□ 1차 도출점 : 지혈까지의 시간 (time to hemostasis)

□ 2차 도출점 : 지혈 후 혈관 합병증 (vascular complication after hemostasis)

- 출혈 [Bleeding (TIMI definition: major, minor, minmal)]

- 혈종 (Hematoma)

- 복막혈종 (Retroperitoneal hematoma)

- 가동맥류 (Pseudoaneurysm)

- 혈관폐색 (Vessel occlusion)

- 박리 (Dissection)

- 근치 수술 (Surgical repair)

- 미주신경 반응 (Vasovagal reaction)

(1) 프로토콜 과정

** 측정 스케쥴

| 변 수 명 | Baseline | Follow Up | |
| --- | --- | --- | --- |
|  |  | Post-Procedure  (1day) | 30 days(±2w) |
| 문 진 | O | O | O |
| 신 체 검 진 | O | O | O |
| CBC ( 총혈구검사 ) | O | O | O |
| 지혈까지의 시간 | O | X | X |
| 출혈, 혈종, 가동맥류 발생 여부 | O | O | O |
| 원위부 혈류 | O | O | O |
| 설문지를 통한 불편도 평가 | X | O | X |

1. **효과 평가기준, 평가 방법**

본 연구는 proof-of-concept trial로 국내기업이 개발한 ezClot이 기존의 다른 지혈법과 비교하여 비슷한 성능을 가지고 있다는 것을 확인하기 위한 목적이다. 지혈지표인 primary endpoint (time to hemostasis)와 출혈 지표로 구성된 secondary endpoints의 발생 빈도를 비교하여 효과를 평가한다.

1. **기존 치료 및 연구와의 차별점**

본 연구에서 사용하는 ezHEMO는 임상에서 널리 사용되고 있는 HemCon® pad와 거의 동일한 디자인을 가지고 있음. 지혈시간을 단축할 목적으로 두께를 늘리고, 시술자의 편의를 도모하기 위한 몇가지 디자인을 개선한 것 외에는 HemCon® pad와 임상적으로 중대한 차이점은 없음.

1. **연구대상자의 이익과 위험**

본 연구는 새로이 개발된 의료기기에 대한 산학연구로서, 본 연구에 참여하는 연구대상자에게는 지혈기구가 무상으로 제공될 예정임. 한편 본 연구에 참여함으로 인해 기대되는 추가적인 위험은 거의 없다고 판단됨. 다만 아직 임상을 통해 증명되지 않은 의료기기이므로, 과민반응 등의 부작용이 발생하지 않는지 특히 주의 깊게 관찰할 필요가 있겠음.

1. **중지∙탈락 기준**

A. 심각한 부작용의 발생

B. 환자가 강력히 치료 중단을 원할 때

C. 지혈 패드를 사용하면서 과민반응을 보이는 경우

1. **부작용을 포함한 안전성의 평가기준, 평가 방법 및 보고 방법**

본 임상시험 참여 중에 발생하는 모든 중대한 이상 반응은 연구 시험 내용과 관계없이 이 사실을 알게 된 시점으로부터 48시간 이내에 책임 연구자에게 반드시 보고되어야 하며, 각 분기별로 IRB에 보고되어야 한다. 연구에서 정의한 TIMI major bleeding, 사망, 및 과민 반응 외에도 신체적 기능이나 활동, 신체 구조에 영구적이거나 상당한 손상이나 장애가 발생한 경우, 입원치료가 필요하거나 입원기간의 연장이 필요한 경우, 선천성 기형 또는 이상을 초래하는 경우, 사망을 초래할 수 있는 의학적 사건이 발생한 경우도 중대한 이상반응에 해당한다.

연구자는 환자의 안전을 위해 정기적으로 부작용을 분석하며, 예기치 않은 부작용이 발생하였을 경우 IRB에 보고한다.

1. **자료안전성 모니터링 계획(DSMP)**

임상시험이 KGCP에 따라 실시되고 시험자료가 국내외에서 인정될 수 있도록 하기 위해서 필요 시 모니터링 및 점검을 실시한다. 책임 연구자 주관 하에 매 1년마다 모니터링을 시행하며, 모니터링 시에는 연구자 입회 하에 기록원부와 대조 검토 및 증례 기록이 완전하고 명확한 지 확인한다. 연구자는 어느 때든지 모니터 요원이나 점검자에게 협조해야 한다. 모니터링 결과 중대 이상반응이나 예상하지 못한 문제, 계획서 미준수 등의 문제가 발생하는 경우 IRB에 보고하며, 그 기한은 본원 IRB 규정에 따른다.

관혈적 검사에 따른 안전성 지표인 입원 기간 중 TIMI major bleeding 발생을 모니터하여, 유의미한 발생이 있따고 판단되는 경우 연구의 지속, 변경, 중단 여부를 평가한다.

- 이상약물/의료기기반응보고 : fetal/life-threatening) : 사망이나 생명을 위협하는 사례는 7일

이내 (초기보고) + 8일 이내 (추적보고),

- 이상약물/의료기기반응보고 : not fetal/life-threatening : 그 외의 입원 또는 입원기간의 연장, 지속적인 또는 중대한 불구나 기능저하를 초래, 선천성 기형 또는 이상을 초래, 중요한 의학적 사건, 기타) 사례는 15일 이내

- 안전성 관련 정보 보고 : 6개월에 1회 이상

- 예상하지 못한 문제 보고 : 15일 이내

- 중대한 미준수 사례보고 : 15일 이내

- 사소한 미준수 사례보고 : 1년에 1회 이상

1. **자료 분석 및 통계 분석 방법**

1차 도출점인 지혈까지의 시간은 범주형 변수로 chi square test를 이용하여 비교한다. 2차 도출점은 이분형 변수로서 chi square test로 검정한다. 만약 이들 값이 모수 검정에 적절하지 않은 경우, Mann–Whitney U test 또는 Fisher’s exact test를 통하여 분석한다. P값이 0.05 미만인 경우를 통계학적으로 유의한 것으로 정의한다.

1. **연구수행일정표**

| **추진내용** | **세부추진 일정** | | | | | | | | | | | | | | | |
| --- | --- | --- | --- | --- | --- | --- | --- | --- | --- | --- | --- | --- | --- | --- | --- | --- |
|  | 2015 | | | 2016 | | | | | | | | | | | | 비고 |
|  | 10 | 11 | 12 | 1 | 2 | 3 | 4 | 5 | 6 | 7 | 8 | 9 | 10 | 11 | 12 |  |
| IRB 연구 승인 |  |  |  |  |  |  |  |  |  |  |  |  |  |  |  |  |
| 데이터베이스 구축 |  |  |  |  |  |  |  |  |  |  |  |  |  |  |  |  |
| 환자등록 |  |  |  |  |  |  |  |  |  |  |  |  |  |  |  |  |
| 추적관찰 |  |  |  |  |  |  |  |  |  |  |  |  |  |  |  |  |
| 통계분석 |  |  |  |  |  |  |  |  |  |  |  |  |  |  |  |  |
| 보고서 작성 |  |  |  |  |  |  |  |  |  |  |  |  |  |  |  |  |
| 논문 작성 |  |  |  |  |  |  |  |  |  |  |  |  |  |  |  |  |

1. **연구대상자의 안전보호를 위한 대책**
2. **연구의 윤리성 확보를 위한 기본 방안**

본 연구는 분당 서울대학교병원 IRB의 심의와 승인을 얻은 뒤에 시행되며, 헬싱키 선언 (2013년 개정) 및 ICH-GCP를 준수한다.

1. **연구대상자의 동의 과정**

- 연구대상자에게 설명하고 동의를 취득할 연구자: 연구책임자 및 담당자
- 동의를 제공할 자: 연구대상자 본인
- 연구 설명 과정과 동의 취득 과정 사이의 대기 시간: 1-2일
- 강제 또는 부당한 영향의 가능성을 최소화시킬 방법: 연구 참여는 환자 본인의 자발적인 의사에 의해서만 가능하며, 만일 참여를 원하지 않으신다고 해도 추후의 치료과정에는 어떤 영향도 미치지 않을 것임을 설명
- 연구 설명 과정과 동의 취득 과정에서 연구자가 사용하는 언어: 한국어
- 연구대상자 또는 대리인이 이해할 수 있는 언어: 한국어
- 연구대상자 또는 대리인에게 제공되는 정보와 동의서 서식: 별첨문서

1. **연구대상자의 보상 방안**

본 연구에 참여하는 연구대상자에게는 지혈기구가 제공된다. 이러한 보상은 연구대상자가 임상시험에 끝까지 참여할 것을 조건으로 하지 않으며, 연구를 종료하지 못한 연구대상자에게도 동일하게 제공될 것임.

1. **연구대상자의 개인정보보호 방안**

환자의 의무기록번호 및 병리 번호는 주관연구자의 책임하에 별도의 파일로 보관하며 이를 코드화하여 연구데이터를 통하여 개인 신상 확인이 불가능하도록 관리한다. 또는 연구데이터는 패스워드가 걸린 파일에 저장하여 잠금 장치가 있는 연구실에 보관하도록 한다.

1. **취약한 연구대상자를 포함하는 경우 추가적인 보호조치 방안**

해당 없음.

1. **인체유래물의 보관 및 폐기 방법**

해당 없음.

1. **참고 문헌**

1. Cong X, Huang Z, Wu J, Wang J, Wen F, Fang L, Fan M and Liang C. Randomized Comparison of 3 Hemostasis Techniques After Transradial Coronary Intervention. J Cardiovasc Nurs. 2015.

2. Trabattoni D, Montorsi P, Fabbiocchi F, Lualdi A, Gatto P and Bartorelli AL. A new kaolin-based haemostatic bandage compared with manual compression for bleeding control after percutaneous coronary procedures. Eur Radiol. 2011;21:1687-91.

3. Trabattoni D, Gatto P and Bartorelli AL. A new kaolin-based hemostatic bandage use after coronary diagnostic and interventional procedures. Int J Cardiol. 2012;156:53-4.

4. Politi L, Aprile A, Paganelli C, Amato A, Zoccai GB, Sgura F, Monopoli D, Rossi R, Modena MG and Sangiorgi GM. Randomized clinical trial on short-time compression with Kaolin-filled pad: a new strategy to avoid early bleeding and subacute radial artery occlusion after percutaneous coronary intervention. J Interv Cardiol. 2011;24:65-72.

5. Arbel J, Rozenbaum E, Reges O, Neuman Y, Levi A, Erel J, Haskia AR, Caneti M, Sherf M and Mosseri M. USage of chitosan for Femoral (USF) haemostasis after percutaneous procedures: a comparative open label study. EuroIntervention. 2011;6:1104-9.
